# Associations Between Digitally Measured Sleep Quality and Lateral Step Variability Among Older Adults

**DOI:** 10.1093/geroni/igaf122.522

**Published:** 2025-12-31

**Authors:** Francesca Marino, Minzae Kim, Rhoda Au, Phillip Hwang, Jesse Mez, Mike Alosco, Robert Thomas, Ludy Shih

**Affiliations:** Boston University - Chobanian & Avedisian School of Medicine, Boston, Massachusetts, United States; Boston University - Chobanian & Avedisian School of Medicine, Boston, Massachusetts, United States; Boston University - Chobanian & Avedisian School of Medicine, Boston, Massachusetts, United States; Boston University School of Public Health, Boston, Massachusetts, United States; Boston University - Chobanian & Avedisian School of Medicine, Boston, Massachusetts, United States; Boston University - Chobanian & Avedisian School of Medicine, Boston, Massachusetts, United States; Beth Israel Deaconess Medical Center, Boston, Massachusetts, United States; Beth Israel Deaconess Medical Center, Boston, Massachusetts, United States

## Abstract

Higher step variability is associated with slower gait speed, reduced leg strength, and increased fall risk. Poor sleep quality is an emerging risk factor for worse physical function, but associations between sleep and step variability are unclear. Evidence from objective sleep measures is also lacking, as most studies rely on self-reported measures that may misclassify sleep quality. Therefore, this study aimed to evaluate associations between digitally measured sleep characteristics and lateral step variability among older adults. This study included 72 older adults (mean age: 71 years) who had ≥2 nights of objectively measured sleep (SleepImage ring) and completed a 10-meter preferred speed walk while wearing APDM sensors on their back, wrists, and feet. Lateral step variability, defined as variability of perpendicular deviations of foot placement, was categorized as medial (≤-7.5 cm), minimal (within ±7.5 cm; reference), or lateral displacement (≥7.5 cm). Demographic-adjusted multinomial logistic regression models evaluated cross-sectional associations between sleep and lateral step variability categories. We found that 1% higher sleep fragmentation was associated with 1.06-times odds of step variability ≥7.5 cm (95% CI: 1.01-1.11), while 1% higher sleep stability was linked with 0.95-times odds of variability ≥7.5 cm (95% CI: 0.91-0.99) compared to variability within ±7.5 cm. There were no associations with sleep duration, efficiency, apnea, or oxygen saturation. Better quality sleep, particularly lower fragmentation and higher stability, was associated with lower lateral step variability. Future studies should evaluate whether digital sleep measures can predict individuals at risk for falls or serve as intervention targets for fall prevention.

